# Psychological well-being and factors affecting it after the COVID-19 pandemic

**DOI:** 10.3389/fpsyg.2024.1295774

**Published:** 2024-02-08

**Authors:** Towhid Babazadeh, Saber Ghaffari-fam, Farzaneh Shahnavaz-Yoshanluie, Soheila Ranjbaran

**Affiliations:** ^1^Department of Public Health, Sarab Faculty of Medical Sciences, Sarab, Iran; ^2^Department of Nursing, School of Nursing of Miyandoab City, Urmia University of Medical Sciences, Urmia, Iran; ^3^Education Department of Ajabshir, Ajabshir, Iran

**Keywords:** psychological well-being, COVID-19 pandemic, health locus of control, adults, Iran

## Abstract

**Introduction:**

The COVID-19 pandemic has enormously impacted human activity worldwide, partly due to many governments issuing stay-at-home orders and limiting the types of social interactions citizens can engage in. Hence, this study investigated psychological well-being and factors affecting it after the COVID-19 pandemic.

**Methods:**

A total of 345 participants were recruited in the study. This research was conducted between February and May 2023 in Sarab, East Azerbaijan, Iran. To measure the data, valid and reliable instruments of Goldberg’s General Health Questionnaire (GHQ-28), Multidimensional Health Locus of Control (MHLC), interpersonal support evaluation list (ISEL-SF), and health-protective behaviors checklist instrument were used.

**Results:**

According to the results, health locus of control (*r* = 0.227; *p* < 0.001), social support (*r* = 0.339; *p* < 0.001), and COVID-19 preventive behaviors (*r* = 0.376; *p* < 0.001) were positively correlated with psychological well-being. The strongest correlation was observed between psychological well-being and protective behaviors (*r* = 0.376; *p* < 0.001). In the hierarchical regression model, total, demographic characteristics along with health locus of control, social support, covid-19 preventive behaviors, and history of COVID-19 infection were able to explain 57.4% of the variation in psychological well-being.

**Discussion:**

Public healthcare providers’ and policymakers’ preventive and supportive actions are highly advised for promoting health locus of control and social support in adults after the COVID-19 pandemic. In addition, it is better to include a community’s social and environmental changes.

## 1 Introduction

The world is in the grip of the COVID-19 pandemic, which has infected over 22 million individuals and caused over 780,000 deaths worldwide (Dong et al., 2020). Although COVID-19 mortality has declined in hospitalized patients, it is still high in hospitalized patients, especially in the elderly (Esmaeili et al., 2023). Many countries applied measures to reduce the spread of the COVID-19 disease, which included staying at home, quarantine, business closures, social distancing, restrictions on gatherings (Pokhrel and Chhetri, 2021), and avoiding unnecessary travel (Rezaei et al., 2023).

During the COVID-19 pandemic, more than one-third of the world’s population experienced high levels of psychological distress (Luo et al., 2020). The restrictions to prevent the spread of the COVID-19 disease have placed a heavy toll on people’s psychological well-being (Serrano-Ripoll et al., 2020). The impacts of the COVID-19 pandemic include disruptions in people’s daily lives and social connections and a reduction in the experience of the sense of belonging (Allen and Furlong, 2021; Lim et al., 2021). In addition, the COVID-19 pandemic has had significant psychological effects on different levels of society, such as high levels of anxiety, depression, stress, fear, fear of COVID-19 vaccine uptake, boredom, loneliness, uncertainty, post-traumatic stress symptoms, confusion, anger, and stigma, which are psychological distress signs in patients (Brooks et al., 2020; Duan and Zhu, 2020; Ranjbaran et al., 2023).

“Social support is an individual’s perception or experience of being involved in a social group where people mutually support each other” (Hajli et al., 2015). Social support plays an important role in promoting mental health (Koelmel et al., 2017).

The longitudinal study has strongly pointed to the relationship between social support and mental health outcomes (Rothon et al., 2012). A meta-analysis conducted by Fangjun et al. (2012) has concluded that mental health is related to social support for aged people. In another study, it was seen that there is a difference between genders in terms of emotional support. Emotional support is associated with mental health in women but not in men (Fiori and Denckla, 2012).

Having sufficient knowledge of COVID-19 alone cannot be correlated with preventive behaviors or risk perception (Taghrir et al., 2020). Meanwhile, fear of COVID-19 or its perceived risk has shown a strong association with COVID-19 preventive behaviors (Ahorsu et al., 2020). Factors other than adequate knowledge of COVID-19, which may be psychological factors, predict preventive behaviors of COVID-19. Therefore, it seems necessary to identify other psychological factors related to COVID-19 to help health professionals and health policymakers draw strategies to deal with epidemics during the outbreak of similar diseases effectively. Path analysis of COVID-19 and psychological distress has shown that believing information about COVID-19 is associated with fear of COVID-19, which is related to preventive behavior and psychological distress (Chang et al., 2020). Hence, this study investigated psychological well-being and factors affecting it after the COVID-19 pandemic.

## 2 Methods

### 2.1 Participants

The current cross-sectional study was conducted between February and May 2023 in Sarab, East Azerbaijan, Iran. Using multistage cluster random sampling, participants were recruited from Healthcare Service Centers (HSCs). In the first step, four HSCs were identified as clusters during the initial stage of sampling. In the second step, individuals were chosen at random from the four HSCs based on their health data. Then, respondents were contacted by phone, briefed about the research aims, and invited to participate in the study. Before completing the questionnaire, subjects signed a formal informed consent form. The questionnaire items were completed by participants in a consultation room at the health clinic. Due to the nature of the study questions and the culture of the study population, all interviews were conducted by two trained interviewers to ensure participant comfort. Inclusion criteria for this study included consent to participate and individuals aged 18–65. The exclusion criteria were refusal to participate in the study and failure to complete the questionnaire completely and correctly. The sample size was determined using data from a similar study (Khasareh and Mirtajadini, 2022) and a confidence level of 97.5%, Z = 2.24, SD = 12.534, Mean = 62.04, 328 samples.

### 2.2 Measure

In order to collect data, valid and reliable instruments were used. A brief description of the questionnaire is as follows:

*Demographic information form*: Demographic information includes participants’ age, gender, marital status, occupation status, education level, income status, and number of family members.

*Psychological well-being*: The Goldberg’s General Health Questionnaire (GHQ-28), which includes 28 items and four subscales (Malt et al., 1989), was used to assess psychological well-being. Each questionnaire subscale has seven items in physical symptoms, anxiety/insomnia, social dysfunction, and severe depression. All scale items are rated on a four-point scale (0–3), with a higher score indicating poorer mental health. The Persian version of the questionnaire’s estimated alpha coefficient was 0.93 (Moeini et al., 2008).

*Health locus of control*: The 18-item Multidimensional Health Locus of Control (MHLC) scale established by Wallston et al. (1978) was used to assess the health locus of control. This scale measures people’s health-related beliefs and includes six items about internal HLC (the belief that one’s behaviors and actions determine one’s state of health), six items about powerful others HLC (the belief that powerful others, primarily professionals, determine one’s state of health), and six items about chance HLC (the belief that one’s state of health is determined by chance). Each item was scored on a six-point Likert scale ranging from 1 to 6 (strongly agree). Previous research has found the Persian version of this scale to be valid and reliable (Moshki et al., 2007).

*Perceived social support*: Perceived social support was assessed by the interpersonal support evaluation list (ISEL-SF) (Payne et al., 2012). The psychometric properties of the scales were documented by Ranjbaran et al. (2014). These four subscales are (a) Appraisal Support, which is the perceived availability of someone to discuss personal issues; (b) Tangible Assets Support, which is the perceived availability of material aid; (c) Belonging Support, which is the perceived availability of others with whom one compares favorably, and (d) Self-Esteem Support, which is the perceived availability of others with whom one compares favorably. A four-point Likert-type scale (certainly true, maybe true, probably false, and definitely false; scored 0–3) was used as the tool. An individual respondent’s overall score value could range from 0 to 48, with higher scores indicating greater felt social support.

*COVID-19 protective behaviors*: The checklist is used to investigate the health-protective behaviors (i.e., were you wearing the mask during the pandemic of COVID-19?) against developed COVID-19 consisted of five items in a five-point Likert scale ranging from “never” (1) to “always” (5), ranging from 0 to 20 (Cronbach alpha obtained 0.80) (Salavati et al., 2021).

### 2.3 Data analysis

Depending on how the data were distributed, percentages and frequencies were used for categorical variables, while the mean, standard deviation, median, and quartile deviation were used for continuous variables. The independent sample t-test and one-way ANOVA were used for bivariate comparisons of quantitative variables. The link between health locus of control, social support, and COVID-19 preventive behaviors with psychological well-being is measured using Pearson correlation. To label the strength of the association, for absolute values of *r*, 0–0.19 was regarded as very weak, 0.2–0.39 as weak, 0.40–0.59 as moderate, 0.6–0.79 as strong and 0.8–1 as very strong correlation (Evans, 1996).

A three-step hierarchical linear regression analysis was conducted to determine which variables can predict psychological well-being. Demographic variables made up Block 1. Block 2 came with demographic characteristics, health locus of control, social support, and COVID-19 preventive behaviors. The variables of health locus of control, social support, and COVID-19 preventive behaviors and demographic variables, along with the history of COVID-19 infection, made up Block 3. To determine the percentage of variance characterized by psychological well-being, we assessed the adjusted R2 change following the insertion of each block. Tests for multi-collinearity, normalcy, and significant data points confirmed the assumptions behind regressions. The threshold for significance was fixed at = 0.05. The significance level was set to α = 0.05. All analyses were conducted using SPSS 21.

## 3 Results

A total of 345 individuals agreed to participate in the study, of which 183 (53%) people reported a history of COVID-19. Table 1 shows the demographic characteristics of the individuals as well as their relationships with psychological well-being. The average age of the participants was 30.43 ± 9.17, with the majority being between the ages of 26–35.

**Table 1:** Demographic characteristics of the subjects and their association with psychological well-being.

Variable		*N* (%)	Psychological well-being	*p*-value*
			Mean ± SD	
Age groups (year)	18 to 25	120 (34.8)	49.93 ± 9.12	0.549
	26 to 35	133 (38.6)	50.25 ± 9.30	
	35 and higher	92 (26.7)	51.30 ± 9.51	
Gender	Male	219 (63.5)	50.86 ± 9.21	0.247
	Female	126 (36.5)	49.65 ± 9.39	
Marriage	Single	72 (20.9)	50.73 ± 8.39	0.748
	Married	273 (79.1)	50.34 ± 9.51	
Income (month)	Lower than 2	128 (37.1)	50.08 ± 9.23	0.756
	2 to 5	82 (23.8)	51.06 ± 10.19	
	6 and higher	135 (39.1)	50.35 ± 8.78	
Job	Office Clerk	114 (33.0)	49.64 ± 9.55	0.556
	Laborer	43 (12.5)	51.79 ± 9.89	
	self-employment	32 (9.3)	49. 78 ± 9.69	
	Housewife	156 (45.2)	50.75 ± 8.84	
Education	Under diploma	134 (38.8)	51.56 ± 9.08	0.188
	Diploma	128 (37.1)	49.77 ± 9.17	
	Bachelor and higher	83 (24.1)	49.57 ± 9.68	
Number of family members	Three and less	143 (41.4)	49.99 ± 9.90	0.600
	Four	117 (33.9)	50.33 ± 8.52	
	Five and above	85 (24.6)	51.27 ± 9.24	

*p < 0.05.

The subjects with and without a history of COVID-19 infection were compared based on the study’s significant variables of interest. Differences in psychological well-being, social support, and protective behaviors were statistically significant at the univariate level, as shown in Table 2. Individuals infected with COVID-19 exhibited lower levels of well-being, social support, and protective actions.

**Table 2:** Comparisons of psychological well-being, health locus of control, social support, and COVID-19 preventive behaviors among people with a history of having COVID-19.

Variables	Status	Mean (± SD)	*p*-value*
	Having a history of Covid-19		
Psychological well-being	Yes	43.41 (± 5.17)	0.001**
	No	56.62 (± 7.54)	
Health locus of control	Yes	67.59 (±16.76)	0.111
	No	70.34 (± 15.16)	
Social support	Yes	34.36 (± 6.34)	0.001**
	No	37.87 (± 6.17)	
Covid-19 preventive behaviors	Yes	9.02 (± 3.30)	0.001**
	No	11.74 (± 4.43)	

*Independent samples *t*-test. **p < 0.05.

Table 3 shows the variables of psychological well-being, health locus of control, social support, and COVID-19 preventive behaviors as determined by the Pearson correlation test. According to this test, all variables were positively correlated with psychological well-being (*p*-value ≤ 0.05). The strongest correlation was observed between psychological well-being and protective behaviors (*r* = 0.376; *p* < 0.001).

**Table 3:** Bivariate correlation between psychological well-being, health locus of control, social support, and COVID-19 preventive behaviors.

Variables		1	2	3	4
1 = Psychological well-being	*r*	1			
	*p*	–			
2 = Health locus of control	*r*	0.227*	1		
	*p*	0.001			
3 = Social support	*r*	0.339*	0.105	1	
	*p*	0.001	0.052		
4 = Covid-19 preventive behaviors	*r*	0.376*	0.065	0.245*	1
	*p*	0.001	0.230	0.001	

*Correlation is significant at the 0.05 level (two-tailed).

The hierarchical regression model examines effects of demographic characteristics, health locus of control, social support, and COVID-19 preventive behaviors on psychological well-being. In step 1, demographic characteristics accounted for 2.4% of the variation in psychological well-being (*F* = 1.36; *p*-value = 0.226). In step 2, health locus of control, social support, and covid-19 preventive behaviors explained an additional 23.3% variation in psychological well-being (*F* = 26.241; *p*-value < 0.001). As can be seen in Table 4, health locus of control, social support, and preventive behaviors were the statistically significant predictors of psychological well-being. In step 3, a history of COVID-19 infection was added, which explained an additional 31.7% of the variation (*F* = 247.76; *p*-value < 0.001). Step 3 findings display that history of COVID-19 infection (ß = −0.156; *p*-value = 0.010) was the strongest significant predictor of psychological well-being (β = 0.617; *p*-value < 0.001). In total, demographic characteristics along with health locus of control, social support, COVID-19 preventive behaviors, and history of COVID-19 infection were able to explain 57.4% of the variation in psychological well-being (Table 4).

**Table 4:** Hierarchical linear regression for predicting psychological well-being through demographic characteristics, health locus of control, social support, and COVID-19 preventive behaviors.

Variables	ß	R2 change	F change	SE	*p*-value
Step 1					
Age	0.119	0.024	1.36	0.06	0.226
Gender	0.042			1.15	
Job	0.072			0.56	
Marriage	0.060			1.34	
Education	0.068			0.71	
Number of family members	0.017			0.65	
Step 2					
Age	0.084	0.233	26.241	0.05	0.001
Gender	0.029			1.02	
Job	0.045			0.50	
Marriage	0.030			1.19	
Education	0.081			0.64	
Number of family members	0.035			0.59	
Social support	0.243*			0.07	
Health locus of control	0.165*			0.03	
Covid-19 preventive behaviors	0.311*			0.11	
Step 3					
Age	0.080	0.317	247.76	0.04	0.001
Gender	0.012			0.77	
Job	0.91			0.37	
Marriage	0.007			0.89	
Education	0.050			0.48	
Number of family members	0.003			0.44	
Social support	0.129*			0.05	
Health locus of control	0.100*			0.02	
Covid-19 preventive behaviors	0.142*			0.08	
History of Covid-19 infection	0.617*			0.72	
Total R2	–	0.574	**–**	–	
Adjusted R2	–	0.560	**–**	–	

*p < 0.05.

## 4 Discussion

The COVID-19 pandemic had a significant psychological impact on various levels of society. Hence, this study investigated psychological well-being and factors affecting it after the COVID-19 pandemic. The subjects with and without a history of COVID-19 infection were compared based on the study’s significant variables of interest. The result of the study demonstrated that differences in psychological well-being, social support, and protective behaviors were statistically significant at the univariate level. Individuals infected with COVID-19 exhibited lower levels of well-being, social support, and protective actions. The other study demonstrated that almost 42% of adolescents reported psychological well-being as bad during the pandemic, and 19% of adolescents had depression risk (Jusienė et al., 2022). Fear of COVID-19 was significantly associated with poor well-being (Khan et al., 2021). In confirmation of the results of our study, it was seen that there is a significant relationship between the health status and psychological effects of the COVID-19 pandemic and the psychological well-being status (Maberah and Al-Safasfh, 2020). Social, economic, and government service disruptions have been more visible and shown immediate consequences of COVID-19 on well-being (Zhu and Holden, 2023). According to one study, psychological inflexibility mediated the relationship between COVID-19 stress and psychological well-being (Duckering, 2022). A study showed that health behaviors related to COVID-19 were influenced by social support, altruism, life experience, and cognitive factors (Rezakhani Moghaddam et al., 2022; Brennan-Ing et al., 2023). These findings reveal that infectious diseases and pandemics can affect all aspects of people’s lives.

According to the results of this research, variables of health locus of control, social support, and COVID-19 preventive behaviors were positively correlated with psychological well-being. The strongest correlation was observed between psychological well-being and protective behaviors. The study showed that internal locus of control was associated with positive psychological well-being and relatively better coping with COVID-19 anxiety (Banerjee et al., 2022). Also, another study found that the external locus of control was a strong predictor of COVID-19 conspiracy suspicion (Anderson, 2023). Similarly, the absence of severe emotional and behavioral problems, less sedentary behavior, and a high level of school social capital were factors determining adolescents’ psychological well-being, and low level of physical activity played an important role in school students’ poor well-being (Jusienė et al., 2022). Given this fact, it is important to investigate psychological well-being and factors affecting it during the coronavirus pandemic. Preventive behaviors such as physical activity are the major determinants of psychological well-being pre-and post-COVID (Jusienė et al., 2022). Importantly, public health strategies to improve psychological well-being in adults should aim to simultaneously promote health locus of control, social support, and COVID-19 preventive behaviors.

Based on the results of the present study, demographic characteristics accounted for 2.4% of the variation in psychological well-being. Additionally, health locus of control, social support, and COVID-19 preventive behaviors explained an additional 23.3% of the variation in psychological well-being. As can be seen in Table 4, health locus of control, social support, and preventive behaviors were the statistically significant predictors of psychological well-being. Also, a history of COVID-19 infection was added, which explained an additional 31.7% of the variation, and the history of COVID-19 infection was the strongest significant predictor of psychological well-being. In total, demographic characteristics along with health locus of control, social support, COVID-19 preventive behaviors, and history of COVID-19 infection were able to explain 57.4% of the variation in psychological well-being. The results of our research confirm the decrease in psychological well-being in adults during the pandemic. Thus, healthcare professionals should pay specific attention to health locus of control, social support, and preventive behaviors and provide them with educational programs during pandemic and crisis times. The model can also be suggested for professional interventions to design coping strategies among adults for the challenges of psychological well-being during the COVID-19 pandemic by predictor factors. This research results align with previous studies on a similar topic (Jusienė et al., 2022; Lábadi et al., 2022). The study results implied that psychological flexibility components were important targets for prevention and intervention efforts amid the COVID-19 pandemic (Kroska et al., 2020).

### 4.1 Limitations

This population-based research covered the analysis of several important factors, for instance, intra-individual, inter-relational, public health, and social factors. However, there were limitations in this study. The self-reporting measures used in the study might be biased in responders. In addition, data were collected in a city; thus, the results need to be more generalized to other countries.

## 5 Conclusion

In sum, individuals infected with COVID-19 exhibited lower levels of well-being, social support, and protective actions. Health locus of control, social support, and COVID-19 preventive behaviors were positively correlated with psychological well-being. The strongest correlation was observed between psychological well-being and protective behaviors. Public healthcare providers’ and policymakers’ preventive and supportive actions to implement and steadily sustain the programs are highly advised to promote flexibility, health locus, control, and social support in adults.

## Data availability statement

The raw data supporting the conclusions of this article will be made available by the authors, without undue reservation.

## Ethics statement

The studies involving humans were approved by the Ethics Committee of the Sarab Faculty of Medical Sciences (Ethics Code: IR.SARAB.REC.1401.005) approved this research. The studies were conducted in accordance with the local legislation and institutional requirements. Written informed consent for participation in this study was provided by the participants’ legal guardians/next of kin.

## Author contributions

TB: Data curation, Formal analysis, Methodology, Software, Writing – original draft. SG-f: Investigation, Project administration, Resources, Validation, Writing – review & editing. FS-Y: Writing – review & editing, Validation, Visualization. SR: Writing – review & editing, Conceptualization, Project administration, Supervision, Writing – original draft.
